# Erosive Twiddler’s Syndrome: A Severe Case with Externalization of the Pacemaker

**DOI:** 10.7759/cureus.7458

**Published:** 2020-03-29

**Authors:** Ryan Stuart, Zachary Gilbert, Damian N Valencia

**Affiliations:** 1 Internal Medicine, Kettering Medical Center, Kettering, USA; 2 Internal Medicine, Kettering Medical Center, Dayton, USA

**Keywords:** pacemaker, twiddler's, reeling, ratchet, coiling, erosive, ppm, ts

## Abstract

Implantable pacemakers have been the mainstay of therapy for patients with severely decreased left ventricular ejection fractions and recurrent arrhythmias, among other cardiac pathology. Twiddler’s syndrome (TS) is an uncommon but potentially life-threatening complication of pacemaker therapy, defined as pacemaker malfunction in the setting of device lead dislodgment due to physical manipulation. Traditionally, there are three distinct TS variants (reeling, ratchet and coiling). This case offers evidence of a unique and new variant of TS, severe recurrent erosive subtype with pacemaker externalization.

## Introduction

Implantable pacemakers have been the mainstay of therapy for patients with severely decreased left ventricular ejection fractions, recurrent arrhythmias and other cardiac pathology since their initial implementation in the late 1950s [1]. Shortly after, Twiddler’s syndrome (TS) was first described; it is an uncommon but potentially life-threatening complication of pacemaker therapy [2]. TS is defined as pacemaker malfunction in the setting of device lead dislodgement due to physical manipulation [2]. The majority of these patients, regardless of TS variant (reeling, ratchet or coiling), are diagnosed within the first year of implantation [3]. This case offers evidence of a unique and new variant of TS, severe recurrent erosive subtype with pacemaker externalization, which resulted in multiple pacemaker insertion site adjustments.

## Case presentation

The patient is a 56-year-old man with a pertinent past medical history of non-ischemic dilated cardiomyopathy (left ventricular ejection fraction of 46.4%, recovered from 10%) secondary to uncontrolled permanent atrial fibrillation status post failed direct cardioversion and pharmacologic rate control, requiring atrioventricular node ablation and biventricular pacemaker placement for cardiac resynchronization therapy (October 2017).

Shortly after pacemaker implantation, the patient experienced discomfort with significant pruritus at the insertion site. Persistent picking at the site eventually led to mild cellulitis, which was treated with a course of oral clindamycin and cephalexin. Unfortunately, after total resolution of the soft tissue infection, the patient continued to experience discomfort at the pacemaker site. The patient described an aching, burning, cramping and sharp shooting pain in the left anterior chest wall. Eventually, due to significant persistent symptoms, not improved with physical therapy and pharmacologic therapies, the patient underwent pacemaker pocket revision (March 2018). Again, the patient continued to experience pruritus at the site, persistently picking at the pacemaker daily, leaving large excoriations throughout the anterior chest wall. Over the coming months, the patient was admitted again for anterior chest wall cellulitis, eventually discharged with an oral course of doxycycline and experiencing total resolution of all cellulitic changes. The patient's picking behaviors persisted throughout the year, leading up to presentation at an urgent care center (October 2018) for significant erythema and pain at the pacemaker site. The urgent care physician placed the patient on oral clindamycin, with instructions to follow up with his primary care provider within the coming days to ensure resolution.

**Figure 1:**
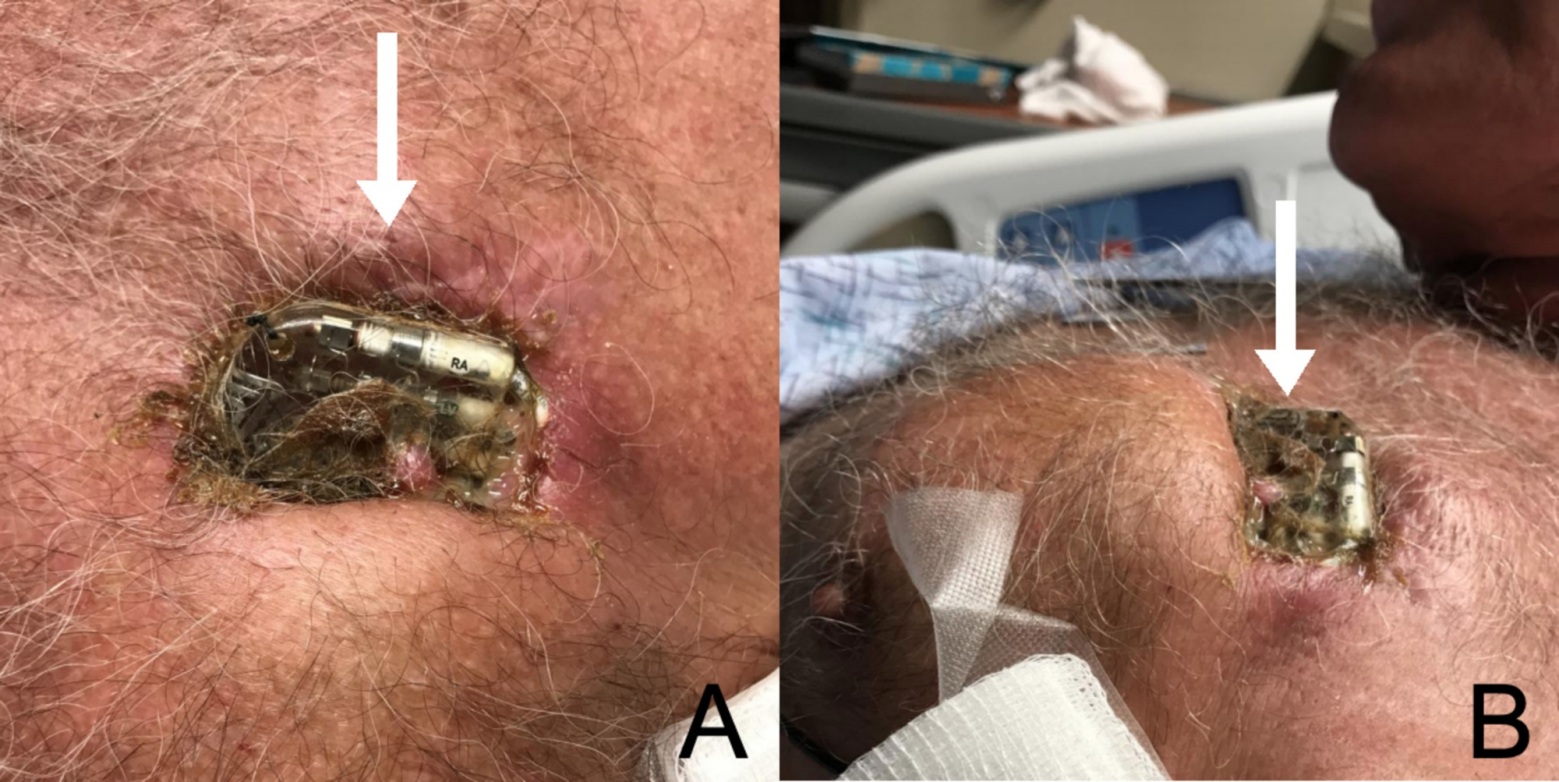
Cutaneous Erosion and Pacemaker Externalization Photograph showing anterior (A) and lateral view (B) of the patients chest wall. Arrows are pointing at the exposed pacemaker.

Soon after, the patient then presented to the emergency department with concerns over a “ruptured boil” and possible exposure of the pacemaker (November 2018). On presentation, the patient reported significant rubor, calor and dolor at the pacemaker site, with intermittent fevers and chills, over the past one month. The patient denied any other symptoms, including chest pain, shortness of breath, palpitations, lightheadedness and syncope. The patient was afebrile and otherwise hemodynamically stable. The patient's cardiac rhythm was paced, with no obvious pacemaker malfunction. Serologic studies only revealed mild leukocytosis. Examination was striking, revealing total cutaneous erosion and pacemaker exposure, with surrounding cellulitic changes and purulent discharge (Figure 1).

**Figure 2:**
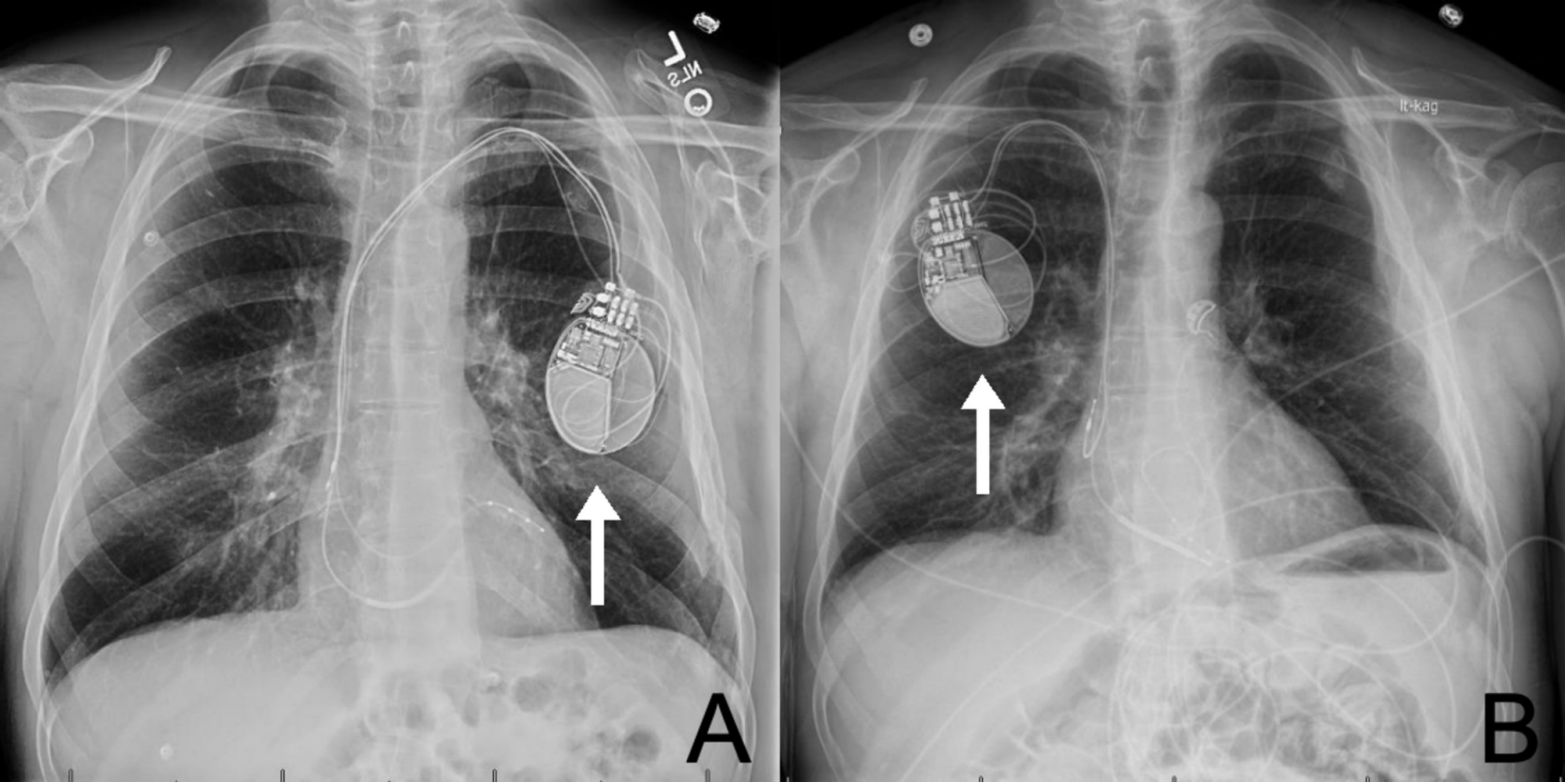
Chest Radiograph Anterior posterior chest radiograph of the patient detailing the original pacemaker site (A) and reinsertion site (B). Arrows are pointing at the pacemaker.

The patient was started on broad-spectrum intravenous antibiotic therapy with vancomycin and piperacillin/tazobactam. Infectious disease and cardiology were promptly consulted. The pacemaker was explanted and replaced in the right anterior chest wall (Figure 2).

Blood and wound cultures returned positive for pan-sensitive Staphylococcus epidermidis. Antibiotic therapy was narrowed to intravenous cefazolin 2 g three times daily, and eventually transitioned to oral cefadroxil 500 mg twice daily for 14 days. The patient experienced a full recovery and was documented as doing well at an outpatient follow-up appointment, although picking behaviors continue and the patient refuses to follow up with psychiatry or psychology.

## Discussion

TS was first described in 1968, only one decade after the first implantable pacemaker [1,2]. Although TS is uncommon, presenting in 0.07%-7% of patients, it is described as a catastrophic malfunction of implantable permanent pacemaker (PPM) therapy and should be considered in all patients [4,5]. As stated above, TS is defined as pacemaker malfunction in the setting of device lead dislodgement due to physical manipulation [2]. In most cases, whether unintentional or deliberate, this is due to the rotation of the pacemaker within the skin pocket causing coiling and/or retraction of the leads. Patients who are elderly, obese, female or have existing psychiatric illness are at increased risk [4,6,7]. The relative size of the subcutaneous pocket compared to the implanted device, PPM or implantable cardioverter defibrillator, is also critical.

Many subtypes of TS have been described in the literature. Reel syndrome is a variant characterized by the winding or reeling of a pacemaker, eventually causing lead displacement and extraction. It is identified by the repetitive rotational movement about the Z-axis. Reel syndrome has typically been associated with increased subcutaneous pacemaker pocket size and repetitive manipulation [8,9]. Ratchet syndrome, like reel syndrome, involves rotational movement about the Z-axis; however, it is suspected that an oscillating movement is implicated in the gathering of pacer leads, as opposed to continuous rotational forces [10]. Others have also described a coiling variant, with rotation about the Y-axis within the pacemaker pocket. Combination of these variants has also been reported [11]. In the case detailed above, lead dislodgement and pacemaker failure were not present. Pacemaker extraction and replacement were primarily due to persistent patient picking of the pacemaker, eventually causing subcutaneous erosion and soft tissue infection. We have termed this severe variant erosive TS. It is important to note that although it is common for pacemaker leads to become extracted during pacemaker manipulation, lead advancement is also possible [12]. TS has also been noted with other implantable devices, like spinal cord and vagal nerve stimulators [13-15]. 

When treating patients with TS, it is important to evaluate for psychiatric conditions, as many correlations have been documented with OCD and other picking disorders [16,17]. Implantable device shape should be considered, as elongated devices have been linked with spontaneous rotation [18]. When re-implantation of a device is necessary, the addition of antimicrobial therapy to the device pouch or envelope may reduce rates of infection [19]. Some have also suggested a dual anchoring technique, along with non-absorbable sutures [20]. 

## Conclusions

TS was initially defined as pacemaker malfunction in the setting of device lead dislodgement, as a result of physical manipulation. It is now known that any accessible subcutaneous implantable device can be affected. Known TS variants (reeling, ratchet and coiling mechanisms) have been well documented throughout the medical literature, and have the potential for occurring in combination. We have presented an additional variant related to persistent pacemaker site picking, which we have termed erosive TS. When evaluating any patient with TS, it is essential to evaluate for comorbid psychiatric conditions, as it has been known to be associated with obsessive-compulsive disorder and other picking disorders. Treatment typically includes device extraction and re-implantation. During re-implantation, additional measures can be taken to reduce recurrence, including dual anchoring and use of non-absorbable sutures, along with antimicrobial device envelopes. 
